# Servant Leadership Stimulates Spiritual Well-Being Through Team Trust in a Female Religious Context

**DOI:** 10.3389/fpsyg.2021.630978

**Published:** 2021-09-03

**Authors:** Innocentina-Marie Obi, Hillie Aaldering, Katalien Bollen, Martin Claes Euwema

**Affiliations:** ^1^Occupational & Organizational Psychology and Professional Learning, KU Leuven, Leuven, Belgium; ^2^Work and Organizational Psychology, University of Amsterdam, Amsterdam, Netherlands

**Keywords:** servant leadership, team trust, team conflict, spiritual well-being, convent

## Abstract

This study investigates how female religious leaders nurture spiritual well-being in religious sisters. Specifically, we examined how servant leadership fosters spiritual well-being [Gifts and Fruits of the Spirit (GFSp)] through, respectively, the mediating role of team trust and reduced occurrence of team conflicts. Quantitative survey data were collected from 453 religious sisters (followers) within a Catholic Women Religious Institute in Nigeria. Using structural equation modeling, results showed that servant leadership is positively related to team trust and negatively related to team conflict. Further findings showed that servant leadership indirectly fosters spiritual well-being: Gifts of the Spirit (GSp), and Fruits of the Spirit (FSp), through the mediating role of team trust, however not through reduced team conflict. Theoretical and practical implications are discussed.

## Introduction

Leaders in religious contexts usually hold responsibilities for the spiritual well-being of followers, particularly when religious leaders and followers live and work 24/7 in religious communities, such as convents. In such a context, trust in each other is the key to well-being, and conflicts threaten both community life and personal as well as spiritual well-being. In this study, we focus on the role of servant leadership in the presence of team trust and team conflict as antecedents of spiritual well-being.

Convents in our study are local religious communities within a Catholic Women Religious Institute, mostly situated in Nigeria, also in other parts of the globe. Convents are of relatively small size (between 2 and 20 or more religious women, with a local community leader). Each local religious community or convent comprises an appointed leader. One of the responsibilities of the leader is to foster the holistic well-being (physical, emotional, moral, intellectual, spiritual, and psychological) of the followers (Obi and Bollen, 2017). Religious sisters strive for holistic well-being, both as an individual and as a community, and spiritual well-being forms an important element of holistic well-being.

Leadership plays a key role in the holistic well-being of individuals in every organization. Specifically, servant leadership, which prioritizes serving followers over and above the leader (Greenleaf, 1977; Eva et al., 2019) has been strongly associated with promoting the needs and well-being (Chiniara and Bentein, 2016; Hoch et al., 2018; Roberts, 2021) of the followers. As servant leadership is rooted in spiritual and moral insights, humility, and spirituality (Greenleaf, 1977; Graham, 1991; Sendjaya et al., 2019), it is even more related to spiritual well-being (Okonkwo, 2015). Servant leadership may not always impact followers directly. Crucial processes such as trust in the leader (Schaubroeck et al., 2011; Van Dierendonck, 2011; Shim et al., 2016), follower need satisfaction (Van Dierendonck et al., 2014; Chiniara and Bentein, 2016), and cooperative conflict management (Wong et al., 2018), have been found to enhance the relating power of servant leadership in business contexts.

While research investigating leadership and trust have extensively focused on the trust of the followers in the leader (Dirks and Ferrin, 2002), we study how servant leaders build the trust of the followers in each other, which in this study is referred to as team trust. In contrast to team trust, which indeed nurtures warm relationships and collaboration, stands team conflict, which may destroy collaboration and cooperation among individuals. Team conflicts between team members occur often and are inevitable, also in convents. While team conflict can be beneficial under specific circumstances (De Wit et al., 2012), their detrimental effects on the well-being of individuals have been highlighted (De Dreu and Weingart, 2003; Bendersky and Hays, 2012; Greer and Dannals, 2017; Römer, 2017). One of the critical factors in curbing team conflicts in organizations is leadership (Römer, 2017; Babalola et al., 2018; Zhao et al., 2019; Obi et al., 2020). Given its priority on the well-being of the followers, we focus on whether and how servant leadership can promote the perceptions of spiritual well-being of the sisters in convents, measured as “Gifts and Fruits of the Spirit” (GFSp), through increased team trust and decreased conflict occurrence.

The current study contributes to the extant literature on servant leadership, team trust, conflict, and well-being in several ways. First, we describe and understand spiritual well-being based on the theological notions of GFSp. Although these notions may enhance organizational effectiveness, they have received little attention in organizational literature. Second, research relating servant leadership to team trust (trust of followers in each other instead of trust in the leader) is scarce. Third, while we know that context plays a crucial role in leadership research (Oc, 2018), much of the research on leadership and organizations takes place in Western or Asian business contexts (Eva et al., 2019). Our study in local religious communities or convents in Nigeria is rather unique. We thus add to extant research on servant leadership, which has rarely been studied in African (Eva et al., 2019; Roberts, 2021) and/or in religious contexts.

In what follows, we describe relevant literature on spiritual well-being based on the GFSp as well as on servant leadership, team trust, and team conflict. We then argue for the hypothesized relationships and describe in the methods section our approach to data collection and statistical analyses. We explain and interpret results to finally discuss the implications for research and practice as well as limitations of our study and suggestions for future research.

### Spiritual Well-Being

Spiritual well-being is a multifaceted construct, including the concepts of spirituality (McClendon, 2015), health, wholeness, and well-being (National Interfaith Coalition on Aging (NICA), 1975; Fisher et al., 2000). In 1975, spiritual well-being was initially defined by the National Interfaith Coalition on Aging (NICA) (1975), as “the affirmation of life in a relationship with (1) God, (2) self, (3) community, and (4) environment that nurtures and celebrates wholeness.” In assessing spiritual health or well-being, there is no specific universally accepted measure (Fisher, 2016; Fisher and Ng, 2017). In fact, Fisher (2015) found about 300 measures of spirituality and spiritual health or well-being used in the last five decades. A dominant measure is the 20-item spiritual health and life orientation measure (Gomez and Fisher, 2003), as well as its shortened version, which is the four-item spiritual well-being index (Fisher and Ng, 2017). Fisher (1998, 2016) defines spiritual well-being as a dynamic state of being, demonstrated by the degree to which individuals live in harmony in four domains, namely personal (meaning, aim, purpose, and life values), communal (morality, culture, and religion), environmental (awe and wonder), and transcendental (God, something or someone beyond the level of human, optimal concern, and transcendent reality). In this study, we build on these four domains of spiritual wellbeing- personal, communal, environmental, and transcendental, by investigating spiritual well-being based on the perceived presence of GFSp in a group. We focus on the experience of spiritual well-being within the team (that is, among religious sisters in local religious communities), acknowledging the communal element of the definition which would imply that one can hardly experience high spiritual well-being if interdependent others do not share in this experience.

Prior studies showed that spiritual well-being serves as a protective measure for individuals facing serious emotional stress (Hardiman and Simmonds, 2013; Oman and Bormann, 2015) and enhances job satisfaction and resilience among the youth undergoing difficult times (Tejeda, 2015). Moreover, demonstrating spiritual well-being enhances influence and satisfaction, whereas poor exhibition of spiritual well-being engenders depression and unfulfillment in business school professors of religious universities (Harajli, 2021). These results emphasize the need for spiritual well-being in individuals, but have not directly examined the spiritual well-being of groups. While most studies on spiritual well-being have focused on business contexts, specifically among health institutions, research in religious organizations (Okonkwo, 2015; Del Castillo et al., 2020) is limited.

### Gifts and Fruits of the Spirit

Spiritual well-being in various religious traditions has been defined not only as a state of mind but also as a practice, demonstrating virtues. In the Christian tradition, spiritual well-being is generally referred to as a relationship with God and with human beings, which may be perceived from the Gifts and the Fruits of the Holy Spirit, deeply rooted in the Old and New Testament of the Bible (GSp and FSp, see Table 1). Emphasis on the GFSp can also be found in theological research (Keating, 2000; Bouchard, 2002; Ten Klooster, 2019; Anton, 2020; Richert, 2020) and in the influential *Summa Theologiae-Prima Secundae and Secunda Secundae* of Thomas Aquinas *(ST. 1–2, Q. 68; 2, Q. 68–70)*. The Gifts of the Spirit (GSp) are principles of moral or ethical behaviors (virtues) and the FSp are the concrete actions or behaviors formed by virtues and inhibited by vice (Ten Klooster, 2019). Thus, GSp, also the principles or values of moral life, aid the FSp to be espoused (Ten Klooster, 2019; Anton, 2020). Hence, we posit that both “Gifts” and “Fruits” of the Spirit represent the spiritual well-being of individuals, such as religious sisters, and can also be found in other religious traditions, and among diverse individuals in their daily events of life. We focus on the GSp and the FSp as virtues and behaviors that sustain the spiritual and moral life of the individual and the groups (Catechism of the Catholic Church, 2002) (CCC, nr. 1830–1832). Hence, spiritual well-being in this study encompasses the transcendental (relationship with God) and existential (relationship with self, with the community and with the environment) life of religious sisters in convents.

**Table 1:** Spiritual well-being: GFSp.

**Gifts of the spirit**	
*Wisdom*	Enables individuals to respond to humans with charity and patience; and to perceive the wonders of the natural environment, the historical events, and the uncertainties of human life as profoundly meaningful, with the Transcendent being (e.g., God) at work in them.
*Understanding*	Empowers human beings to begin to trust as a result of what they understand through contemplation rather than what they know, hence order their lives toward the final end.
*Counsel*	Ability to act appropriately in the matters of faith, ethics, and morals, and thus, enhancing right judgment.
*Fortitude*	Inspires individuals with the courage, and consistency of will to stand firm without fear in the face of physical or spiritual difficulty, and avails them coping strategy for survival in times of poverty, and hardship, and hence endure a long trying moments.
*Knowledge*	Instills in people the capacity to make quick, and effective discernment between what is good, and what is not good, thus choose the right path.
*Piety*	Motivates people to respect others, to perform their responsibilities willingly, and to see other humans, not as competitors in the life's journey and struggle, but as co-equals.
*The fear of the lord*	Fosters individuals' desire not to detach themselves from the transcendent reality, thus maintaining a “filial” rather than a “servile” fear; a gift of love of the transcendent (God).
**Fruits of the spirit**	
*Love*	The highest virtue indicating pure love of God and neighbor. Love (Agape) embodies self-sacrifice, compassion, goodwill, charity, self-giving, and unconditional love.
*Joy*	A state of being undisturbed by unconstructive events of life. Joy depicts inner tranquility, and communal state of elation and celebration.
*Peace*	Fosters inner tranquility in humans in times of chaos and confusion.
*Patience*	Ability to endure in times of suffering, and bear the imperfections of other humans when provoked by them.
*Kindness*	Enables individuals to be compassionate toward others, and give them more than they really deserve; a selfless behavior, fairness, and consideration of others, helping others, and sharing in other's joys and sorrows.
*Goodness*	Inspires consistent ethical behavior.
*Gentleness*	The ability to forgive hurts, show compassion, and mercy to people rather than giving way to easy outburst of anger.
*Faithfulness*	Deep trusting and trustworthy behavior. Faithfulness spurs individuals to be reliable, dependable and loyal.
*Self-control*	Inspires moderation, and temperance, in individuals, such that they resolutely keep excessive wants, and desires in check, inner humility and simplicity.

*Two spiritual well-being components as assessment tool for spiritual well-being*.

The GSp are identified in the book of Isaiah: “*And the Spirit of the Lord shall rest upon him. The spirit of wisdom and understanding, the spirit of counsel and might, and the spirit of knowledge and the fear of the Lord and his delight shall be in the fear of the Lord (Isaiah 11:2)*.” The seven GSp thus include wisdom, understanding, counsel, fortitude, knowledge, piety, and fear of the Lord.

Wisdom enables individuals to order the created and the spiritual world properly; to respond to humans with love (charity) and patience (Richert, 2020). Wisdom fosters the ability of individuals to perceive the wonders of nature, the historical events, and the uncertainties of human life as entirely meaningful; and to participate in God's presence in the world (Fiddes, 2021) and ordered by God. Wisdom further depicts the ability of an individual to know how to live well, and to actually live good life that is essential and valuable for wellbeing (Grimm, 2015, 2017). Hence, the core of wisdom is the fear of the Lord (Longman, 2021) evident in relationship with God or in love of God and neighbor.

Understanding supersedes natural reason that focuses on what is visible to human senses. It is the gift of tolerating others, showing empathy and compassion, and can potentially foster collaboration.

Counsel inspires individuals to assess correctly how best to behave in certain circumstances (Richert, 2020). It fosters in individuals the ability to act appropriately in matters of faith and morals and enhances right judgment.

Fortitude captures the extent to which individuals are enabled to confront rather than succumb to fear, and to demonstrate the consistency of will when confronted with physical or spiritual difficulties (Richert, 2020). It enhances the coping strategy of people toward survival when confronted with poverty, loss, and hardship.

Knowledge encourages people to know and to choose the right path. With the gift of knowledge, individuals are inspired to make quick and effective discernment between what is good and what is not (Hardon, 2020).

Piety refers to reverence or loyalty (without compulsion). It inspires respect for parents and significant others and enables individuals to carry out their daily (spiritual) duties willingly. Piety enables people to see other humans not as competitors in the journey of life, but as coequals (Hardon, 2020), thereby enabling individuals to identify with others in times of suffering (Keating, 2000).

Fear of the Lord avails individuals the desire not to separate themselves from the transcendent (God). It is a “filial fear,” rather than a “servile fear.”

The Gifts of the Holy Spirit may be viewed as the convergence between morality and the spiritual life (Bouchard, 2002), and these Gifts serve to complement and perfect human activity, indicating a high level of spiritual well-being. Additionally, they help the FSp to be enacted (Ten Klooster, 2019). While the GSp are virtues, the FSp are actions that these virtues produce (Richert, 2020), or the ethical behaviors that individuals or groups display. Yet, both the GSp and the FSp are also referred to as virtues and can be stimulated, developed and advanced in convents for efficient community life.

### The Fruits of the Spirit

The FSp are found in the letter of St. Paul to the Galatians: “*But the Fruit of the Spirit is love, joy, peace, patience, kindness, goodness, faithfulness, gentleness, self-control*” (Gal 5:22–23) (see Table 2).

**Table 2:** Results of structural equation modeling of standardized indirect effects with non-significant effects.

**Indirect effects**	**Hypothesis**	**Standardized estimate**
SL → Team trust → Gifts of the spirit	4a	43^***^
SL → Team trust → Fruits of the spirit	4b	0.46^***^
SL → Team conflict → Gifts of the spirit	4c	-0.02ns
SL → Team conflict → Fruits of the spirit	4d	-0.01ns

*SL, Servant leadership;*

** *p < 0.01,*

*** *p < 0.001, ns, non-significant*.

Love (charity) indicates the pure love of God and neighbor (Agape). Love is altruistic, self-sacrificial, and unconditional love that enables individuals to put the good of others above their own good while expecting no reward (Richert, 2020).

Joy depicts a state of being undisturbed by unconstructive events in the present life (Richert, 2020). Joy is deeper, more tranquil, and more stable than merely worldly emotional happiness that lasts momentarily.

Peace depicts tranquility, calmness, and serenity in the soul of an individual despite the anxiety and uncertainties in the experience of life. Peace enables people to be at peace with themselves in the time of chaos and confusion (Ten Klooster, 2019). Indeed, peace is an important Fruit of the spirit that depicts integrity and justice, and is an indicator of health and wellbeing that enables individual's feeling of holistic safety.

Patience depicts the ability of an individual to endure, tolerate, and bear the imperfections and inadequacies of others with compassion through the acknowledgment of their own imperfections and the need for (God's) mercy and forgiveness. It inspires people to be patient in times of suffering (Ten Klooster, 2019).

Kindness inspires the willingness of individuals to give to other people beyond what they deserve (Richert, 2020). Kindness is an active show of goodness in daily life through selfless behaviors, fairness, and compassion, by considering others, helping others, and sharing joys and sorrows, both in good and hard times (Ten Klooster, 2019).

Goodness may be perceived as consistently embracing good behavior and doing what is morally right, even at the higher expense of the reputation, success, status, and fortune of an individual for the sake of good and for the well-being of others. Goodness has been considered as innate to human existence (Floyd, 2015), and it is thereby staying true, genuine or authentic to one's characteristic nature, which may be perceived as a virtue. Hence, virtues have been indicated as closely knotted to goodness and community (Tjeltveit, 2003) of individuals that exhibit goodness or good deeds.

Gentleness depicts being compassionate and showing mercy to people rather than giving way to an easy outburst of negative emotions such as anger and revenge. A gentle individual expresses meekness and humility, and will not insist on having his/her own way. Bocarnea et al. (2018) perceive gentleness as appropriate use of power.

Faithfulness is the ability of an individual to constantly live life in a good way; trusting others humans and being trustworthy, reliable, dependable, and loyal. It has been viewed also as demonstrating authenticity (Bocarnea et al., 2018). Faithfulness indicates being reliable, dedicated, dependable and loyal towards oneself, and others, and towards the environment and the transcendent.

Self-control implies moderation, temperance, and resolutely keeping the wants and desires under control, to attain excellent goals through upholding virtuous behaviors (Zell and Baumeister, 2013).

In sum, the GSp accommodate the enactment of the FSp, which together form indicators of spiritual well-being. Research focusing on the Gifts and Fruits of the Spirit as indicators of spiritual wellbeing is rare.

Both the GSp and the FSp have been recognized as indicators of spiritual well-being in theological literature, and these claims have been substantiated with research. Previous scales have assessed parts of the GSp such as wisdom (Tredget, 2010), as well as FSp, with a specific focus on Christian virtues, leadership, and employee performance (Bocarnea et al., 2018). Research has shown that the Fruit of the Sprit, such as self-control is a virtue and the moral strength that enables moral behavior (Baumeister and Exline, 1999, 2000), which improves community harmony. Recent research has included altruistic love and faith among spiritual measures, which serve as spiritual practices to transform a dysfunctional organization into a functional work environment (Dean, 2020). Till now, however, no measure to our knowledge incorporates all GFSp to measure spiritual well-being. This is surprising, given that the GFSp fit with the four-dimensional definition of spiritual well-being, which is as follows: the extent to which individuals live in harmony with the self, community (humanity), the natural environment, and the transcendent reality; and additionally measures how this is accomplished in behavior. We build on and extend previous research on spiritual well-being by incorporating all the seven Gifts and nine FSp in our measure. Importantly, the previously mentioned measures for spiritual well-being typically focus on the individual level. However, of old, spiritual well-being also is a group construct, reflecting the well-being of the community, and the strong bond between the members. Metaphors such as “we are all parts of one body” are lived strongly. Particularly also in a collectivistic society, such as our research context, religious women in convents express a strong collective relationship and sisterhood. Thus, we further expand existing measures by investigating the GFSp as a group phenomenon based on individual perceptions.

For religious leaders, it is imperative to foster these GFSp to ensure spiritual well-being in their teams. In this study, we examine whether and how servant leadership may add to nurturing and advancing GFSp in religious sisters thereby stimulating their spiritual well-being in convents.

### The Role of (Servant) Leadership

Servant leadership is referred to as an “other-oriented approach to leadership manifested through one-on-one prioritizing of follower individual needs and interests, and outward reorienting of their concern for self toward concern for others within the organization and larger community” (Eva et al., 2019, p. 112). The “other-oriented” nature of servant leadership may include the committed effort of the leader to enhance the spiritual well-being of the followers. Greenleaf (1977) introduced servant leadership into an organizational context, describing a servant leader as a servant, with a major focus on serving followers.

Servant leadership has been distinguished from other leadership behaviors such as transformational leadership due to its focus on serving the needs and well-being of followers (Van Dierendonck, 2011; Eva et al., 2019), and through several measures, servant leadership has been shown to promote the positive outcomes and well-being (Eva et al., 2019) of followers.

Liden et al. (2015) distinguished seven key dimensions of servant leadership: (1) Emotional healing: caring about the personal and individual problems and well-being of the followers. (2) Creating value for the community: involvement in building community and inspiring followers to be active in the community. (3) Conceptual skills: demonstrating competency in solving work-related problems and understanding the goals of the organization. (4) Empowering: entrusting followers with responsibility, autonomy, and decision-making. (5) Helping followers grow and succeed: helping followers attain their maximum potential and succeed in their vocation. (6) Putting subordinates first: prioritizing meeting the needs of the followers before attending to their own needs. (7) Behaving ethically: Demonstrating honesty and trustworthy behaviors and serving others as a role model of integrity. It is clear that servant leaders promote trust of the followers in each other and their holistic well-being, including spiritual well-being. We utilized the model of Liden et al. (2015); which is a notable rigorous measure (Eva et al., 2019).

Servant leadership shows a wide range of beneficial follower outcomes at individual, group, and organizational levels, as evidenced in reviews (Parris and Peachey, 2013; Eva et al., 2019), and meta-analyses also in explaining more variance than related leadership behaviors in important constructs including trust in the leader (Banks et al., 2018; Hoch et al., 2018; Lee et al., 2020; Legood et al., 2021). Servant leadership promotes trust in the leader (Shim et al., 2016), develops strategies for managing conflicts (Jit et al., 2016), and is rooted in spirituality (Sendjaya et al., 2019). Servant leaders play a crucial role in developing and maintaining productive team processes. Below, we elaborate on the potential role of servant leaders in building team trust and curbing the occurrence of team conflicts. First, we explore the relation of servant leadership with Ubuntu, an African leadership paradigm.

*Ubuntu: African roots of servant leadership*. This study focuses on Nigerian religious women. Nigeria, a Sub-Saharan West African context, is characterized by high collectivism, high humane orientation, and high-power distance (Hofstede, 2001; House et al., 2002). The indigenous Nigerian/African setting is rooted in community, and in leadership that cares about the well-being of the people. This leadership paradigm is expressed in the African concept of Ubuntu or Unhu, a human relational concept that promotes transformation, peace, and unity of purpose (Mawere and Van Stam, 2016). Ubuntu further depicts harmony and trust, love and compassion, honesty, forgiveness, integrity, goodwill among individuals, and also places important value on human beings. The Ubuntu African leadership philosophy depicts a community-oriented paradigm where people show interconnectedness and respect for others in peaceful coexistence. In this respect, Ubuntu shows strong similarities with the principles of servant leadership.

### Hypotheses Development

#### Servant Leadership and Team Trust

Trust has generally been defined as “the willingness of a party to be vulnerable to the actions of another party based on the expectation that the other will perform a particular action important to the trustor, irrespective of the ability to monitor or control that party” (Mayer et al., 1995, p. 712). Two trust dimensions are central in this trust definition, which are first, willingness to accept vulnerability to trust others (propensity to trust), and second, perceived trustworthiness in terms of expecting good deeds from the person trusted (trustee). While for Mayer et al. (1995), trustworthiness factors are built in three dimensions, namely: ability, benevolence, and integrity, and this study adds predictability as the fourth dimension following Adams et al. (2008).

Benevolence is the extent to which a team member is perceived to be truly an embodiment of care, interest, protection, and concern about other team members. Integrity is the degree to which a team member is considered to be honest, truthful, and fair (just), such that what the person says aligns with what the person does. Predictability refers to the extent that the behavior of a team member is consistent or reliable, and the ability or competence depicts the extent to which a team member displays valuable skills and proficiencies that enable team effectiveness (Adams et al., 2008).

Trust is a key process of collaboration in religious community life. Here, trust could be perceived as the way a religious sister is willing to be vulnerable to the behaviors of other religious sisters based on the confidence that other religious sisters would exhibit benevolence (protect the other), predictability (transparency), integrity (honesty), and competence in the daily religious community life and work.

Servant leadership is founded on the premise that followers grow as (human) persons to become healthier, wiser, freer, and more autonomous and more capable to become servants to others (Greenleaf, 1977; Parris and Peachey, 2013). We argue that servant leadership empowers followers. To this extent, servant leaders coach, mentor, and support followers and encourage open and effective communication as well as information sharing (Sousa and Van Dierendonck, 2016). This relational behavior of a servant leader encourages followers to develop a willingness to trust each other and to exhibit trustworthy behaviors. Servant leadership gives rise to benevolence and integrity through care, compassionate love, empathy, and by listening (in a nonjudgmental way) to their followers (Van Dierendonck and Patterson, 2015; Coetzer et al., 2017). Followers may replicate these serving behaviors toward their team members, which can instigate team trust. This assertion follows prior research that the decision of a team member to trust their teammates is often based on how much benevolence (concern, care), ability, and integrity a teammate exhibits (Mayer et al., 1995). In his theoretical model, Van Dierendonck (2011) insisted that servant leadership fosters follower outcomes by enhancing a key positive psychological climate—trust. Based on the foregoing reasoning, we propose:

**Hypothesis 1a**: Perceived servant leadership is positively related to team trust.

### Servant Leadership and Team Conflict

Conflict is a part of everyday life. Team conflicts occur between two or more individuals or parties when at least one of these parties feels irritated, frustrated, or obstructed by the other (Van de Vliert, 1997). Typically, conflict behaviors are the responses that individuals in conflict exhibit to those frustrations (Elgoibar et al., 2017), which may either escalate the conflict or help to mitigate the existing conflict.

Although team conflict is not always destructive, several meta-analyses show most team conflicts to have a harmful impact on the health, psychological well-being, and performance of individuals, a relationship that is often affected by the way conflicts are managed (Bendersky and Hays, 2012; De Wit et al., 2012; Elgoibar et al., 2017; Greer and Dannals, 2017). Conflicts challenge teamwork, including religious community living (Obi et al., 2020), and important processes to mitigate conflict occurrence among team members, including leadership has been consistently highlighted.

A key role of leadership is intervening in conflicts to solve these in a positive way (Römer et al., 2012; Römer, 2017; Babalola et al., 2018; Zhao et al., 2019; Obi et al., 2020). Servant leadership could reduce the occurrence and escalation of conflict among team members by promoting ethical behaviors (Liden et al., 2015), connecting followers to openly share information, and communicating in a constructive and effective way. Studies have indeed shown that servant leadership develops positive and efficient conflict management strategies (Jit et al., 2016), and relates strongly to positive and cooperative conflict management behaviors (Wong et al., 2018; Obi et al., 2020), which are relevant for curbing conflicts. In addition, servant leaders could apply their conceptual skills to detect whether something is going wrong within the team (Liden et al., 2015) or community. Further, servant leadership has been presented as a favorable leadership behavior in crises, given its emphasis on serving the needs of the followers over and above those of the leader (Firestone, 2020). Based on the preceding reasoning, we hypothesize:

**Hypothesis 1b**: Perceived servant leadership is negatively related to team conflict.

### Team Trust and Spiritual Well-Being

To enhance spiritual well-being among sisters, team trust could play a significant role. As indicated above, team trust reflects the willingness of an individual (trustor) to believe that another person (trustee) is genuinely benevolent, predictable, and shows integrity and competence (Mayer et al., 1995; Adams et al., 2008). An individual (trustor) who believes that others (trustees) genuinely have her/his interests in mind (benevolence), and acts fair, honest, and just (integrity), would demonstrate virtues (GSp). Moreover, when a religious sister (trustee) demonstrates competence in daily functions, and a wide range of knowledge about religious community life, others will be inspired to live the GSp. These trustworthy behaviors would likely nurture deeper bonding with oneself (personal), with other humans or the community (communal), with the environment and with the transcendent, and thus stimulate spiritual health. Moreover, trustworthy behaviors strengthen wisdom, which enables individuals to harness their relationship with other humans (Richert, 2020), with oneself, with the environment and with the transcendent. It also enables individuals to perceive others with dignity, value, and respect. Individuals in trusting relationships would then begin to find deeper meaning in whatever that happens around them (see also Table 1). Trust in the competences or in the abilities of the team members and in their integrity, and predictability, could further stimulate their gift of understanding and other Gifts of the Spirit (see Table 1). Thus, to consistently stimulate the GSp among religious sisters, practicing trust in each other is relevant. Based on this reasoning, we propose the following:

**Hypothesis 2a**: Perceived team trust is positively related to the GSp.

### Team Trust and FSp

We argue that the key dimensions of team trust, namely benevolence (care and looking out for others), integrity (fairness and honesty), predictability (consistency and faithfulness to plans), and competence (trust in abilities of an individual and effective communication) (Adams et al., 2008) could together promote the FSp. For example, a team member or members after making a mistake, would expect other team members to be benevolent (and compassionate) by offering forgiveness (Tjosvold et al., 2016). This benevolence (forgiveness) experienced by a team member or team members, would inspire FSP- love (charity), joy, peace, patience, kindness, goodness, faithfulness, gentleness, and self-control (see Table 1). The FSp can hardly be developed in an organization without committed relationships with others. In other words, the Fruits of the Spirit can hardly be developed in local religious communities without sisters' committed trust relationships and connecting with each other. Hence, the trust that religious sisters have in each other would likely fuel the exhibition of the Fruits of the Spirit, thereby stimulate spiritual wellbeing in convents. Following this line of thought, we expect:

**Hypothesis 2b**: Perceived team trust is positively related to the FSp.

### Team Conflict and Spiritual Well-Being

We already mentioned the most negative impact of team conflict on the well-being and performance of individuals as well as on the collaborative teamwork (Bendersky and Hays, 2012; De Wit et al., 2012; Tjosvold et al., 2019). In conflicts, people get insecure, lack trust, and lose confidence in other people and in themselves and hence, issues get more challenging and problematic and conflicts escalate. Similarly, research has shown that individuals in conflicts often become overly emotional, angry, and are unable to see the viewpoints of others (Bollen and Euwema, 2015; Elgoibar et al., 2017). They become narrow-minded such that it seems difficult either to live out the GSp—wisdom, understanding, knowledge, and piety, or bear FSp, which are peace, patient, or to show love for the other person, as people feel hurt. Following this line of thought, we formulate:

**Hypotheses 3**: Perceived team conflict is negatively related to (***H3a***) GSp and (***H3b***) FSp.

### Servant Leadership and Spiritual Well-Being—The Mediating Role of Team Trust

Servant leadership behaviors are capable of advancing spiritual well-being of their followers. Several studies have consistently associated servant leadership with such virtues as compassionate love, humility, forgiveness, and interpersonal acceptance (Van Dierendonck and Nuijten, 2011; Van Dierendonck and Patterson, 2015; Jit et al., 2017; Sousa and Van Dierendonck, 2017). Others include ethical and moral behaviors such as honesty, trust, integrity, and spirituality (Liden et al., 2008; Sendjaya et al., 2019). These virtuous and moral potentials associated with servant leadership suggest a strong link between servant leadership and the spiritual well-being of the followers. While servant leadership may not always foster the well-being of the followers directly, critical team processes such as team trust could serve as an underlying psychological mechanism to mediate a proposed relationship.

This study posits that servant leadership could foster spiritual well-being (GSp) through stimulating team trust among their followers. As argued previously, the follower-centered premise of servant leadership, and its ethical behaviors, namely honesty and trust (Liden et al., 2015), would inspire the trust of followers among each other. Van Dierendonck (2011) indicated that the person-oriented attitude of a servant leader makes way for safer and stronger relationships within the organization. In precarious situations, team members are convinced that they could rely on other team members to be fair (e.g., Adams et al., 2008), as they believe that their team members have their best interest at heart, which may foster a conducive environment for spiritual well-being. We posit that servant leadership would positively inspire the trust of the team members in each other through its virtuous or ethical behaviors and emotional healing (Liden et al., 2015). Consequently, team trust will in turn advance the spiritual wellbeing of followers through inspiring the GSp.

We further propose that servant leadership fosters FSp through the mediating role of team trust. When team members trust each other and are perceived to be trustworthy, they visibly portray behaviors depicting that they could be benevolent, transparent, reliable, and competent. These trustworthy behaviors will likely enhance their expressions of love, joy, peaceful behaviors, patience, kindness, goodness, faithfulness, gentle behaviors and self control in relating with self, with the community, the environment and the Transcendent. Since rigorous reviews and meta-analytic evidence have concurred that servant leadership behavior strongly influences the well-being of their followers through mediating variables, such as trust in the leader (Parris and Peachey, 2013; Eva et al., 2019; Lee et al., 2020), we posit that servant leadership will likely promote spiritual well-being through building trust among team members. Based on the foregoing reasoning, we hypothesize:

**Hypothesis 4**: Team trust mediates the relationship between perceived servant leadership and (***H4a***), GSp, and (***H4b***) FSp.

### Servant Leadership and Spiritual Well-Being—The Mediating Role of Team Conflict

While conflicts often impact well-being in a negative way, servant leadership could curb conflict occurrence by encouraging followers in conflict to discuss their issues in a constructive way (Römer et al., 2012; Wong et al., 2018; Obi et al., 2020), and conversely promote psychological well-being of the followers (Rivkin et al., 2014). Servant leadership has been qualitatively found to reduce team conflict by initially diagnosing conflict issues and by humane and participative approaches (Jit et al., 2016).

When followers experience low levels of conflict as a consequence of such servant leadership behaviors, they can indeed manifest their gifts of wisdom and understanding and can demonstrate counsel, fortitude, knowledge, piety, and love of God in charity (fear of God) toward each other, rather than fear of punishment, misunderstanding, hatred, and demonstrating discouragement, as a result of conflict. When followers experience lower levels of conflicts or no conflicts at all, or feel confident to be able to handle conflicts, then they can further exhibit love (agape: Divine Love), joy, peace, patient, kindness, modesty, fidelity, and goodness, rather than hate, negative energy, discord, impatience, unkind attitudes and behaviors, infidelity to community life, and greed, as a result of lack of self-control, which conflicts could instigate. Thus, FSp can materialize within teams in the absence of disruptive conflict. Based on this reasoning, we hypothesize:

**Hypotheses 4**: Team conflict mediates the relationship between servant leadership, and (***H4c***) GSp, and (***H4d***) FSp.

## Materials and Methods

### Sampling and Data Collection

We applied a quantitative survey research approach and assessed the survey either online or with paper and pencil.

The data for this study were collected from religious sisters living and working in local religious communities of a Catholic Women Religious Institute (CWRI) founded in Nigeria. The general leadership team of the Religious Institute provided us with written permission to conduct our research, and all participants signed informed consent. A total of 777 female participants received the questionnaire survey. Respondents were basically Nigerians coming from 166 out of 221 local religious communities within a Religious Institute at the time. The majority of our participants lived and worked in Nigeria and also in other parts of Africa (*n* = 407), Europe, and North America (*n* = 46). The researcher visited the research site to distribute the questionnaires to be filled with paper and pencil. Two planned seminars were used to distribute the questionnaires and collect the data. The final sample consisted of 453 respondents who completed the questionnaire (response rate 58.3%), of which 56 were completed online and 397 *via* paper and pencil. Paper and pencil were much more used for data collection since a good number of the respondents did not have sufficient access to the Internet. Research was conducted between 2016 and 2017.

All participants were women and were all religious sisters. Instead of age, we used the number of years of the participants in the religious profession: The mean age of profession was 18.92 (SD = 10.14), ranging from 1 to 51 years in religious life. The average tenure in convents was 4.77 (SD = 3.06). Participants held different educational backgrounds, with most of them holding a bachelor's degree (30.9%), 26.5% a master's degree, and 3.5% a doctoral degree. Their fields of work varied widely with education as the most dominant professional area of work (60.3%), followed by medical and health (17.4%), accounting (7.7%), and pastoral ministry.

### Measures

#### Servant Leadership

Servant leadership was measured using the seven-item scale developed by Liden et al. (2015). Sample item for the leaders, adapted to fit the religious context: “My local superior puts my best interest before her own.” Cronbach's α is 0.66.

### Team Trust

In order to measure team trust among religious sisters, a 20-item version of the “Trust in Teams Scale” by Adams et al. (2008) was utilized and adapted to suit the religious community context, assessing four dimensions of team trust. The subscales include the following: benevolence (five items), sample item is “My sisters look out for me” with Cronbach's α of 0.83; integrity (five items), sample item is “My sisters honor their words,” Cronbach's α is 0.90; predictability (five items), sample item is “My sisters are reliable,” Cronbach's α is 0.81, and competence (five items), sample item is “I have confidence in my abilities of my sisters,” Cronbach's α is 0.80. Cronbach's α for the general scale is 0.94.

### Team Conflict

Team conflict was measured with 13-items, nine items were adapted from the team conflict scale as developed by Jehn and Mannix (2001) measuring relationship conflict (three items), task conflict (three items), and process conflict (three items): The other four items measuring status conflict were adapted from Bendersky and Hays (2012). Sample items include the following, for relationship conflict, “sisters in my community experienced emotional conflict;” (α = 0.63); task conflict, “sisters in my community frequently had disagreements about the task we are working on” (α = 0.77); process conflict, “sisters in my community often disagree about who should do what” in my community (α = 0.85); and status conflict, “sisters in my community frequently took sides during conflicts (α = 0.79).” Cronbach's α for the combined team conflict scale is 0.91, which offered us ample reason to utilize these four conflict types as one team conflict construct (see also the descriptive Table 3).

**Table 3:** Means, SDs, correlations, and scale reliabilities of studied variables.

**Measure**	**M**	**SD**	**1**	**2**	**3**	**4**	**5**
1. Servant leadership	4.80	0.87	(0.66)				
2. Team trust	5.12	0.93	0.47^***^	(0.94)			
3. Team conflict	3.85	1.27	−0.20^***^	−0.36^***^	(0.91)		
4. Gifts of the spirit	5.55	0.85	0.28^***^	0.58^***^	−0.16^**^	(0.88)	
5. Fruit of the spirit	5.51	0.87	0.29^***^	0.63^***^	−0.23^***^	0.78^***^	(0.92)

** *p < 0.01;*

*** *p < 0.001; Cronbach's α coefficients are written in parenthesis on the diagonal*.

### Spiritual Well-Being—GSp and FSp

Items for GFSp scale were generated from the review of relevant literature (Bouchard, 2002; Fisher, 2016; Fisher and Ng, 2017; Bocarnea et al., 2018; Ten Klooster, 2019). The current measurement of spiritual well-being contains a total of 16 items comprising GSp (seven items) and FSp (nine items). The key difference between the present spiritual well-being scale and other scales measuring spiritual well-being is the particular emphasis on both the GSp and the FSp as indicators of spiritual well-being. Note that our operationalization focuses on the Gifts and Fruits perceptions within the team, rather than those experienced and expressed by individual members. These items were constructed by the researcher in collaboration with the authors. The response format was on a seven-point Likert scale ranging from “1 = strongly disagree” to “7 = strongly agree.” Participants were asked to indicate to what extent each of the statements corresponded to their experience of spiritual well-being in their local religious communities. Sample item for the GSp reads: “The sisters have understanding and are considerate toward other sisters,” Cronbach's α = 0.89. Sample item for the FSp reads: “We form a joyful community,” Cronbach's α = 0.92.

### Strategy of Analysis

Regarding the new scale for assessing spiritual well-being GFSp, an exploratory factor analysis (EFA) using the principal component analysis (PCA) on 16 items with oblimin rotation in SPSS version 26 was conducted. This resulted in a two-factor model. The Kaiser–Meyer–Olkin (KMO) measure confirmed the adequacy of the sample to be used for the analysis: KMO, 0.94, which Hutcheson and Sofroniou (1999) referred to as “marvelous.” All KMO values for individual items were above the acceptable limit of 0.5 (Field, 2013). The two factors in combination explained 61.25% of the variance. The items that cluster on the same factor suggest that factor 1 exemplifies the nine FSp, while factor 2 typifies the seven GSp (see Table 4).

**Table 4:** Exploratory factor analysis (EFA) results for GFSp.

	**Factors**	
**Item**	**Fruits**	**Gifts**
The sisters… are wise and prudent		**0.75**
… are understanding and are considerate toward other sisters		**0.51**
… are able to counsel or to give guidance to other sisters		**0.76**
… are resilience or determined		**0.84**
… are knowledgeable and searching for growth		**0.72**
…are pious and devoted		**0.67**
… express their love of god in charity		**0.51**
We form a loving community	**0.57**	
We form a joyful community	**0.59**	
We share our sorrows together	**0.56**	
We form a peaceful community	**0.87**	
Sisters are patient and accepting toward each other	**0.87**	
We are a kind community	**0.69**	
We are a forgiving community	**0.88**	
We are a faithful community	**0.51**	
We are a modest community	**0.42**	
Eigenvalues	8.57	1.23
Percentage of variance	53.53	7.72

*Extraction Method: Principal Component Analysis.*

*Rotation Method: Oblimin with Kaiser Normalization.*

*Only loadings over 0.40 appear in bold*.

We utilized structural equation modeling (SEM) to examine the adequacy of the overall model, which enabled us to test simultaneously, our proposed hypotheses. The analysis was conducted with the use of R software, version 3.3.3 with the lavaan package (Rosseel, 2012) version 0.6–1.1132 as well as lavaan survey package (Oberski, 2014). To evaluate the model fit, we applied several goodness-of-fit indices such as the comparative fit index (CFI), the Tucker–Lewis index (TLI), the standardized root mean-square residual (SRMR), as well as the root mean square error of approximation (RMSEA). The CFI and TLI values up to 0.90 and above are acceptable for a reasonable fit. The SRMR values below 0.08, and RMSEA (0.06–0.08) for a reasonable fit (Hu and Bentler, 1999) are acceptable.

The latent constructs, servant leadership, GFSp were constructed with the use of their individual items. As recommended by Little et al. (2013), team trust and team conflict were made out of their respective dimensions or parcels, rather than using the individual items.

Some of the participants in this study live together and belong to the same team (local religious community), and this implies they rated, the same leader, and the same team. Since these observations are dependent rather than independent, we used the lavaan survey to observe the teams as clusters (Oberski, 2014). The use of lavaan survey package in R enhances estimation of our concepts over the clusters, without explicitly modeling the effect of the clusters or teams themselves, as we focus not on the team but the individual.

## Results

### Preliminary Analyses

Table 3 displays the descriptive statistics including the means, standard deviations, reliabilities, and intercorrelations among studied variables. All variables are correlated in the expected directions. Cronbach's alpha for the scales are shown between parentheses at the diagonal. Before testing the hypotheses, we conducted confirmatory factor analyses (CFA) in which we tested two different models to ascertain the dimension of the constructs under study. We constructed measurement model 1, which is a measurement model where the two different constructs representing spiritual well-being, GSp and FSp, loaded on one latent spiritual well-being construct. We compared this first model to measurement model 2, which was based on the five latent variables discussed in the theoretical section above: servant leadership, team trust, team conflict, GSp, and FSp. Both measurement model 1 and measurement model 2 were modified according to the specifications of modification indices.

Measurement model 1 (χ^2^ = 989.808, *df* = 427, CFI = 0.90, TLI = 0.89, RMSEA = 0.05, SRMR = 0.06) generated a good fit. Measurement model 2 (χ^2^ = 888.195, *df* = 423, CFI = 0.92, TLI = 0.91, RMSEA = 0.06, SRMR = 0.05) yielded better goodness-of-fit indices. The four-factor measurement Model 1, although a good fit, did not produce an acceptable TLI cut off criteria (0.90–0.95) (Hu and Bentler, 1999), and so, was not fit for this study. The five-factor model (measurement model 2) made an excellent fit, suggesting that the five constructs are distinguishable and most of the variances could be explained due to different constructs.

### Main Analysis

**Figure 1:**
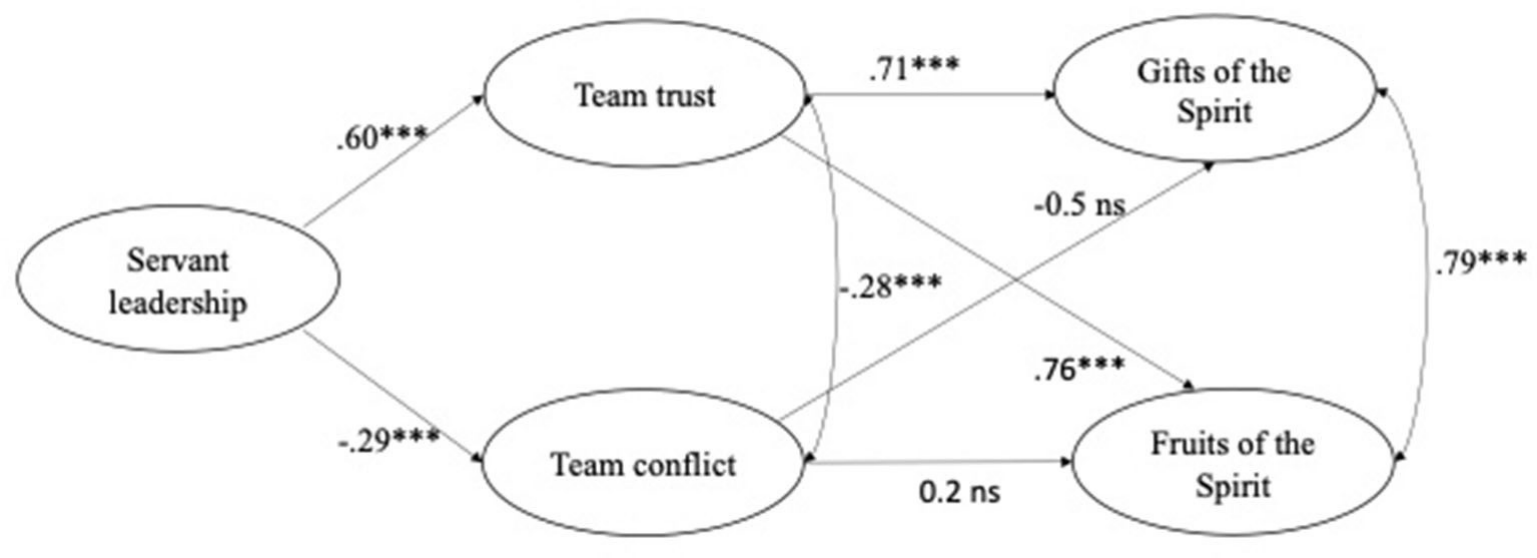
The hypothesized mediation model with SEM parameter and path estimates. ***p < 0.001.

To test the proposed hypotheses, we designed all the hypothesized relationships into a single model (see Figure 1). This was to enhance the simultaneous analysis of the intervening pathways and to ensure a better estimate of the way concepts relate to each other. The structural model produced an excellent goodness of-fit-indices: (χ^2^ = 888.195, *df* = 423, CFI = 0.92; TLI = 0.91, RMSEA = 0.06, SRMR = 0.05). Mediation analysis was conducted to establish whether or not there was an indirect effect (Hayes, 2009; Zhao et al., 2010). To test mediation simultaneously is preferable to conduct them separately (Zhao et al., 2010).

The SEM analysis confirmed H1a, which is servant leadership had a strong positive relationship with team trust (γ = 0.60; *p* < 0.001). Additionally, and as expected, servant leadership was found to be negatively related to team conflict (γ = −0.29; *p* < 0.001) (H1b). Further, team trust was found to be positively related with both (H2a) GSp (γ = 0.71; *p* < 0.001) and (H2b) FSp (γ = 0.76; *p* < 0.001), confirming Hypothesis 2. Against our expectations, team conflict had no significant relationship with either (H3a) GSp (γ = −0.07; *p* = 0.16 ns) or (H3b) FSp (γ = 0.02; *p* = 0.73 ns), rejecting Hypotheses 3a and 3b. The Sobel test revealed a significant indirect effect of team trust, between servant leadership and both the GSp (standardized estimate = 0.43; *p* < 0.001) (H4a) and FSp (standardized estimate = 0.46; *p* < 0.001) (H4b), supporting H4a and H4b, respectively. Given the absence of a relationship between team conflict and GFSp, there was no indirect effect of team conflict between servant leadership and both GSp (standardized estimate = −0.02; *p* = 0.16) (H4c) and FSp (standardized estimate = −0.01; *p* = 0.73) (H4d). Thus, H4c and H4d were not supported in this study.

Notably, while we did not include the direct effect and the total effect in the hypotheses, given our need for indirect effects, these were calculated. All direct effects were non-significant, and thus, were not shown in the final structural model to enhance clarity. When considering only the direct effects, there was no significant relationship between servant leadership and neither the GSp (γ = 0.07, *p* = 0.53 ns) nor the FSp (γ = −0.09, *p* = 0.29 ns). Concerning team trust, the total effect (combining the direct effect, and the indirect effect of the mediating variable-team trust) between servant leadership and GSp was significant (standardized estimate = 0.38, *p* < 0.001). Also, the total effect (combining the direct, and the indirect effects of the mediating variable- team trust) between servant leadership and FSp was also significant (standardized estimate = 0.37, *p* < 0.001). Regarding team conflict as a mediator, the total effect (combining the direct and the indirect effects of the of the mediating variable, team conflict) between servant leadership and the GSp (standardized estimate = 0.07, *p* = 0.37) and FSp (standardized estimate = 0.09, *p* = 0.27) were non-significant.

## Discussion

The current study investigated the direct and indirect relationships between servant leadership and team trust, team conflict, and spiritual well-being in the team and GFSp in religious communities in Nigeria. We found servant leadership to promote team trust (here, trust among religious sisters) directly and spiritual well-being indirectly through fostering team trust. While servant leadership is associated with reduced team conflict occurrence, this was found not to be related to spiritual well-being. These findings show the importance of servant leadership and the development of trust to stimulate and enhance spiritual well-being within the team.

### Theoretical Implications of Study

This study contributes to advancing knowledge in five key ways. First, a direct relationship between servant leadership and team trust is in line with the theories of Greenleaf (2002) that trust is the foundation of servant leadership. This result goes beyond the dominant literature on the trust of followers in leadership (Dirks and Ferrin, 2002; Schaubroeck et al., 2011), and underlines the role of trust in team members. Our finding confirms prior research highlighting trust and positive behaviors of leaders as triggers of the virtuous behaviors of the followers (Malingumu et al., 2016; De Carlo et al., 2020).

Second, we established a direct negative relationship between servant leadership and team conflict. Through relational qualities including good communication skills that foster emotional healing and skills related to community building, servant leaders prevent team conflict from occurring. This finding adds to previous research, indicating that servant leadership behaviors, including humane and participative approaches, could reduce conflict (Jit et al., 2016). By extension, our finding supports the theory that servant leadership could be an excellent leadership behavior in crisis (Firestone, 2020) including team conflicts.

Third, the fact that team trust mediates the relationship between servant leadership and spiritual well-being, GFSp in the team, supports the theory of Van Dierendonck's (2011) on the role of the psychological climate of an organization, such as trust, that could explain the servant leadership–outcome relationship. Team trust is highly emphasized in this study, shedding light on the fact that the four key dimensions of team trust, benevolent, integrity, predictability, and competence (Adams et al., 2008) could together stimulate spiritual well-being in the team, as long as the leader exhibits serving behaviors.

Fourth, the current study introduced the GFSp as indicators of spiritual well-being in the team. While prior organizational studies are beginning to explore virtues such as humility and compassionate love (Van Dierendonck and Patterson, 2015; Sousa and Van Dierendonck, 2017), recent research presented an extensive measure of the FSp in association with leadership (Bocarnea et al., 2018). No research we know of has introduced the GFSp as a measure for spiritual well-being.

Finally, this study was conducted in convents of a Catholic Women Religious Institutes in Nigeria, a West African context. Organizational psychological research has focused on business contexts in the USA and on Asian contexts (see Roberts, 2021). To our knowledge, this is the first study investigating female servant leadership and the implications on team dynamics in such a context and adds to the understanding of leadership and group development in religious communities.

Next to these contributions, our findings also raised some questions. In our testing, we found that team conflict does not mediate the relationship between servant leadership and spiritual well-being. This is surprising, also given the existing and expected direct relations between servant leadership, conflict, and GFSp. These results suggest that team trust is a stronger driver in this relationship, as we used a double mediator design (Hughes et al., 2018).

Trust in team members seems more relevant to religious sisters than focusing on team conflicts. This finding is relevant given that when leaders entrust followers with crucial responsibilities, followers may begin to exhibit trustworthy behaviors in such responsibilities, also toward their team members. Moreover, trusting that team members (religious sisters) could be caring, reliable, transparent, and competent and rating them so, indicates that team members are more interested and more focused in trusting each other than in looking out for conflicts. Further, research has shown that conflict occurs less in an atmosphere of trust (Rousseau et al., 1998). This is worth further study, exploring the interplay between team trust and team conflict, particularly in the context of religious organizations and small communities such as convents.

### Practical Implications

Local community leaders need to be attentive to stimulating trust in the relationships of team members. This can be done by engaging in servant leadership behaviors. Specifically, local community leaders might encourage religious sisters to share information openly and to discuss and reflect on issues together. Indeed, as servant leadership fosters the voice of the followers, followers are more likely to engage in information sharing among themselves (Lee et al., 2020), through which they can handle their conflicts better.

Building team trust among followers is a viable way to stimulate spiritual well-being in religious communities. Leaders and organizations need to see the relevance of servant leadership in encouraging effective interpersonal relationships, collaboration, and honest dialogue among followers. Stimulating spiritual well-being in convents implies advancing growth in wisdom, understanding, counsel and fortitude, knowledge and piety, and fear of God (love of God); as well as enhancing selfless love, joy, and peace, patient, kindness, and goodness, generosity, faithfulness, and modesty or self-control; (see also Table 1). To adequately enhance these virtues (GFSp) in local religious communities demands advancing a servant leadership and a team trust culture and climate.

We, therefore, underline leadership training in religious institutes to be designed in line with the ideas underlying servant leadership and with a strong focus on how to build and stimulate trust among team members (religious sisters). When selecting leaders at all levels of the religious organization, it is needed to pay attention to those individuals that are trustworthy themselves and possess trust building potentials.

Besides training for trust building, selecting competent and potential servant leaders can further promote trust and spiritual well-being – living out the GFSp. For example, research has indicated that with regard to personality traits, training alone could be quite limited, and inadequate, as it can hardly change the steady personality of some individuals. In this regard, it is unlikely that self-centered, authoritarian, and narcissistic people would become relational, person-oriented, sensitive to the needs of others, or learn servant leadership behaviors (Eva et al., 2019) instantly. Our study highlights the importance of (s)electing, training, and coaching those local community leaders and other sisters who show qualities toward advancing servant leadership behaviors and team trust or trust building tendencies for stimulating spiritual wellbeing- GFSp of religious sisters.

### Limitations of Study and Suggestions for Future Research

A key potential limitation of this study is the cross-sectional nature of the data, which inhibits drawing causal conclusions. We believe that the direction of the relationships is not crucial for understanding the need to promote servant leadership, team trust, and spiritual well-being. However, future studies could apply longitudinal and experimental studies to establish causal relations.

Whereas, research on the trust of followers in leadership (Dirks and Ferrin, 2002), and also in servant leadership studies (Joseph and Winston, 2005) has garnered much interest in the past decades, research on team trust is mostly lacking (Lau and Liden, 2008; Braun et al., 2013; Boies et al., 2015). Consistent with the measure for team trust (Adams et al., 2008) and based on our empirical findings, it would be relevant to take this into account in future studies, more specifically, trust among team members.

Moreover, while the concept of servant leadership naturally resonates with spiritual values, it would be important to examine how other leadership styles, such as transformational leadership, may facilitate (or inhibit) the development of spiritual well-being from the perspective of GFSp. Transformational leadership has also been shown to predict team trust (Braun et al., 2013; Chou et al., 2013) and as such might be a likely leadership behavior further foster spiritual well-being too.

Our study is the first to our knowledge to measure spiritual well-being using the GFSp. However, the GFSp have been recognized as indicators of spiritual well-being in theological culture in diverse ways, and these claims have been substantiated with research. Previous measures have also assessed parts of the Gifts and Fruits of the Spirit in various ways to measure spiritual well-being (Gomez and Fisher, 2003; Fisher, 2010, 2011, 2016). Our scale is an expansion of these previous scales. As the goal of our study is not to validate this scale, further research is needed to make the next steps and test for convergent, discriminant, and predictive validity, as well as to test the context-specificity of the scale.

With regards to the seemingly low Cronbach's α values, while the Cronbach's α of the servant leadership scale was surprisingly low, this could perhaps be explained by the specific population that we investigated and/or by slight modifications we made to the items to fit the population. Finally, given the unique context of this study, the results may not be generalized to other contexts. Future research could build on our model in other contexts to enhance its generalizability.

## Conclusion

Our study investigated the direct and indirect effects of servant leadership on team trust, team conflict, and spiritual well-being by means of a questionnaire survey in local religious communities in Nigeria. Results showed team trust to mediate the relationship between servant leadership and spiritual well-being, namely: GFSp as experienced in convents. Further results showed that servant leadership curbs team conflict occurrence in convents. Our results highlight the importance of trusting cooperation and the role of servant leadership in building team trust stimulating spiritual well-being: the experience of GFSp. As such, servant leadership is highlighted in this study to be specifically beneficial and appropriate for Catholic Women Religious Institutes.

## Data Availability Statement

The datasets used for this research article are not likely to be available to the general public until the end of the ongoing research. However, accessing the datasets would be on request through the corresponding author.

## Ethics Statement

The study was conducted according to the guidelines of the Declaration of Helsinki, and reviewed and approved by the KU Leuven Doctoral Committee. As such, further review was not necessary according to the University's ethical guidelines. Written and signed informed consent was received from all the participants.

## Author Contributions

All authors designed the study. I-MO collected and analyzed the data and wrote the first draft. HA, KB, and ME provided feedback. All authors contributed to manuscript revisions, as well as approved the final submission of the current version.

## Conflict of Interest

The authors declare that the research was conducted in the absence of any commercial or financial relationships that could be construed as a potential conflict of interest.

## Publisher's Note

All claims expressed in this article are solely those of the authors and do not necessarily represent those of their affiliated organizations, or those of the publisher, the editors and the reviewers. Any product that may be evaluated in this article, or claim that may be made by its manufacturer, is not guaranteed or endorsed by the publisher.
